# P03 Antimicrobial stewardship nurses—the story so far

**DOI:** 10.1093/jacamr/dlae217.007

**Published:** 2025-01-23

**Authors:** Naomi Fleming, Susan Bowler, Laura Ahearn, Shumirai Matereke, Fiona Marshall

**Affiliations:** NHS England, UK; Nottingham University Hospitals Trust, Nottingham, UK; Northern Care Alliance NHS Foundation Trust, UK; West Suffolk Hospital NHS Foundation Trust, UK; NHS England, UK

## Abstract

**Background:**

The UK AMR national action plan (NAP), ‘Confronting antimicrobial resistance 2024 to 2029’ (www.gov.uk), highlights the workforce as key to tackling AMR, stating ‘We will further embed, and will require, the completion of appropriate IPC and AMS training for all health and social care workers and students, to support implementation of best practice for IPC and AMS in their setting and, for specialist posts, to provide career pathways to promote skills retention and succession planning. Role-specific training will be critical to empowering all staff groups to implement IPC and AMS principles according to their specific roles and responsibilities’. A national AMS nurses’ group was set up in June 2022 with 6 members; there are now 16 members across the UK and Republic of Ireland working primarily in the acute sector. The group holds monthly meetings offering peer support and shares AMS intervention successes. AMS is also pertinent to generalist nursing roles: in 2023, the national AMS nursing group and NHSE AMS team set out to promote AMS with nursing colleagues.

**Objectives:**

To provide insight into AMS for general nurses and spotlight the role nursing colleagues can play.

**Methods:**

A national webinar for nursing colleagues was held in IPC week on 18th October, focusing on AMS aligned with 5Rs of medicines administration: (i) right patient; (ii) right medication; (iii) right dose; (iv) right route; and (v) right time. The session was introduced by Professor Charlotte MacArdle, deputy chief nursing officer on IPC and sustainability, other topics were Start Smart then FOCUS, the role of the nurse in AMS in a hospital setting, QI projects on timely IV to oral switch and the journey to becoming a specialist AMS nurse.

**Results:**

A total of 1060 people from UK registered to attend the webinar from primary, secondary care, the third sector, and various arms-length bodies; 525 joined live. A total of 116 people completed the post event survey; 115 wanted to be kept informed of future events. The webinar scored 4.42/5 for meeting expectations. On average the five presentations were scored as definitely or mostly: interesting: 92%; educational: 90%; and relevant: 75%. Comments were overwhelmingly positive; improvements suggested were more interactivity and inclusion of presentations for community sectors. Examples of attendee actions as a result of the webinar: reach out to AMS nurses; be an antimicrobial steward; promote IVOS; education session on AMS; and participate in AMR groups.

**Conclusions:**

There is a need to embed IPC and AMS as everyone’s job rather than leave it to specialists alone. There is an appetite from the nursing workforce, but further awareness is needed. The new AMS Nursing Platform – Antimicrobial Resistance Programme – FutureNHS Collaboration Platform page advertises events, shares resources and educational materials. Another webinar is being held in October 2024 to capitalize on the success of the first webinar including presentations in community settings. This webinar will be aligned with the six domains of the antimicrobial stewardship competencies for undergraduate nurse education.

